# Using Point of Care Rapid Cortisol Measurement During Adrenal Venous Sampling in Primary Hyperaldosteronism

**DOI:** 10.3390/diagnostics14232692

**Published:** 2024-11-29

**Authors:** Hadas Rabani, Mohammad Sheikh-Ahmad, Robert Sachner, Sawsan Yosefia, Michal Yeiches, Limor Chen-Konak, Clara Henig, Balsam Dakwar, Anan Shalata, Katya Jovanovic, Ilana Rosenblat, Monica Laniado, Ibrahim Matter, Leonard Saiegh

**Affiliations:** 1 Endocrinology Unit, Bnai Zion Medical Center, 47 Eliyahu Golomb Boulevard, Haifa 31048, Israel; 2 The Ruth and Bruce Rappaport Faculty of Medicine, Technion, Haifa 31096, Israel; 3 Invasive Radiology Unit, Bnai Zion Medical Center, Haifa 31048, Israel; 4 Clinical Laboratories Division, Bnai Zion Medical Center, Haifa 31048, Israel; 5 Department of Surgery, Bnai Zion Medical Center, Haifa 31048, Israel

**Keywords:** adrenal venous sampling, point-of-care, primary hyperaldosteronism, rapid cortisol

## Abstract

**Objectives**: To assess success rates and cost-effectiveness of adrenal venous sampling (AVS) after implementing point-of-care rapid cortisol (RC) testing conducted using a europium nanoparticle-based fluoro-immunoassay in patients with primary hyperaldosteronism. **Methods**: A retrospective review of AVS procedures was conducted at our medical center between January 2016 and June 2024. The primary objective was to compare the success rates of AVS before and after the implementation of the RC testing. Secondary outcomes included a cost–benefit analysis. **Results**: Of 55 AVS procedures, 19 were conducted using RC testing and 36 were in the historical control cohort. The success rates for right vein sampling were 79% and 67%, respectively. Overall, in six (31.5%) patients in the RC cohort, a low RC selectivity index (SI) value, calculated within 10 min, enabled determination of unsuccessful cannulation and need for resampling during the same AVS session. Repeated sampling resulted in successful procedures in two cases (10.5%) and unsuccessful AVS in four cases, nonetheless sparing the need for repeated AVS sessions in 31.5% of cases. Utilizing RC potentially spared 6 patients from repeated AVS sessions, and considering the additional expenses on the RC test, its use afforded cost savings of an average of $1288 per patient. **Conclusions**: We demonstrated the cost-effectiveness of utilizing RC measurement in sparing the need for repeated AVS sessions. RC measurement during AVS enabled identification of correct catheter placement in real time, allowing for prompt decisions regarding the need for additional sampling attempts, thereby reducing subsequent costs of repeated AVS sessions.

## 1. Introduction

Primary hyperaldosteronism (PHA) is the most common endocrine cause of hypertension [1]. The prevalence of PHA varies between 3% and 20% in different hypertensive populations [2,3]. Although PHA entails a higher risk for cardiovascular (CV) and cerebrovascular events than essential hypertension [4], PHA is still underdiagnosed [5]. Moreover, 14 years after the first screening criteria guidelines, less than 1% of patients are screened or receive appropriate treatment [6,7].

Early detection of PHA is essential, as untreated PHA is associated with increased cardiometabolic and renal morbidity and mortality rates [8,9]. Indeed, evidence shows that many of the deleterious effects of PHA, compared with essential hypertension, occur independently of blood pressure regulation, including disproportionately higher rates of cardiovascular disease [10]. These effects result from the impact of elevated aldosterone levels on mineralocorticoid receptors in various target tissues [11].

PHA is classified into two main subtypes, namely, unilateral aldosterone-secreting adenoma and bilateral adrenal hyperplasia (also referred to as idiopathic hyperaldosteronism) [12]. The differentiation between the two subtypes is crucial, as it determines the recommended management. In cases of unilateral PHA, surgical adrenalectomy is the preferred treatment to reduce the risk of CV events, as surgery has been shown to improve CV outcomes and reduce medication dependence [13,14]. In contrast, patients diagnosed with bilateral adrenal hyperplasia will be offered pharmacological treatment, mainly with mineralocorticoid receptor antagonists, as surgery is not expected to resolve hyperaldosteronism [14].

According to the clinical guidelines, most patients diagnosed with PHA require adrenal venous sampling (AVS), as it is the gold standard method to distinguish between unilateral and bilateral aldosterone secretion [15]. Previous studies have demonstrated that imaging alone has low sensitivity and specificity for determining the source of aldosterone secretion [14,16].

Confirming accurate sampling of the adrenal veins is vital in order to achieve clinically significant results. AVS success is defined by the proper cannulation of adrenal veins, determined by the calculation of selectivity index (SI). SI represents the ratio between cortisol in the adrenal vein and that in a peripheral vein. When AVS is performed with cosyntropin infusion, as performed in our study, a ratio of at least 5:1 (SI > 5) indicates successful cannulation [14].

AVS is an expensive and technically challenging procedure, and its success rates differ greatly (52–73%) [15,17,18], mainly due to the complex vascular anatomy, causing difficulties in cannulating the short right adrenal vein. Routine laboratory serum cortisol measurement methods have a slow turnaround (approximately one hour); thus, results of the achieved SI ratio are usually available only retrospectively, after the procedure has ended. In cases of failed sampling, the patients should be offered a repeated AVS attempt in order to achieve meaningful results, which entails additional costs and is burdensome for the patient. Additionally, if the second AVS also fails and the source of secretion cannot be determined, then the patient may be offered pharmacological treatment, potentially missing the opportunity for definitive and clinically more beneficial surgical intervention if the aldosterone secretion is indeed unilateral. In other cases, the patient might undergo surgery that ultimately proves to be unnecessary [12,14].

Intraprocedural cortisol measurement enables timely SI calculation, shortly after venous sampling. A pooled analysis on the usage of intraprocedural cortisol measurement across 11 studies has shown significant improvement in successful bilateral sampling using this technique [19]. Using a point-of-care (POC) device for RC measurement, performed bedside in the catheterization room, precludes the need for transporting the samples, thus shortening the measurement turnaround time and reducing procedural duration. Previous studies demonstrated the efficacy of POC-RC assays in allowing real-time feedback on sampling adequacy [15,17,18,20,21,22].

This retrospective study has examined AVS success rates before and after the implementation of europium nanoparticle-based POC-RC immunoassay in our institution. Moreover, we assessed the procedure’s cost effectiveness by performing a cost–benefit analysis.

## 2. Methods

We conducted a retrospective analysis of AVS procedures performed at our medical center from January 2016 to June 2024. The primary objective was to compare the success rates of AVS before (using a historical control cohort) and after implementation of the POC-RC testing in September 2022. Secondary outcomes included procedure duration, cost, risk for adverse events, and need to repeat AVS.

Patients included in this study were diagnosed with PHA through documentation of a high aldosterone-to-renin ratio, followed by a positive saline suppression test in applicable cases, in accordance with the Endocrine Society Clinical Practice Guidelines [14]. An abdominal CT scan was performed in all cases to assess adrenal gland morphology and venous anatomy. All patients included in this study were non-pregnant individuals aged 18 years or older. The selection criteria were identical for both the RC group and the historical cohort.

Clinical data were collected from the medical records, including patient demographics, biochemical confirmation of PHA, history of hypertension, antihypertensive medications, serum potassium levels, CT findings, AVS data, and surgical pathology.

This study was approved by the institutional ethics committee. As this study involved a retrospective analysis of data obtained for clinical purposes, patient consent was not required.

### 2.1. AVS Procedure

Patients were hospitalized one day prior to the AVS procedure. Upon admission, serum potassium levels were measured and corrected, if necessary. They received a continuous cosyntropin infusion of 50 µg/h, initiated 30 min before the procedure, and continued until AVS completion. Adrenal veins were sampled sequentially, beginning with the right adrenal vein, and inferior vena cava blood samples were drawn immediately after each adrenal sampling as reference peripheral samples. Patients were observed for two hours after the procedure to monitor for any immediate complications. All included AVS procedures were performed by the same interventional radiologist and under similar conditions, both in the RC group and in the historical cohort.

AVS success was determined by calculating SI for each side, defined as the ratio of adrenal vein cortisol to peripheral cortisol. The sampling was considered successful when the SI value was greater than 5, using the traditional laboratory Cobas cortisol measurements. Those criteria are consistent with the recommendations from the 2016 Endocrine Society clinical guidelines on hyperaldosteronism for AVS conducted with continuous cosyntropin infusion [14].

The lateralization index (LI) ratio comparing aldosterone to cortisol ratio of the dominant adrenal vein to the non-dominant one was used to differentiate between unilateral or bilateral aldosterone secretion. LI ≥ 4.0 indicated unilateral, and LI < 3.0 indicated bilateral secretion. Ratios between 3 and 4 were considered indeterminate. Those thresholds are consistent with the 2016 Endocrine Society clinical guidelines, with sensitivity of 95% and specificity of 100% for the detection of unilateral secretion [14]. Patients with unilateral secretion who agreed to surgical treatment underwent laparoscopic adrenalectomy. The pathological examination of the resected adrenals included staining for CYP11B2 (aldosterone synthase), identifying regions of aldosterone production.

### 2.2. Rapid Cortisol Evaluation

The RC measurement was conducted using the iChroma II (Boditech©, Seoul, Republic of Korea) test assay. The compact test analyzer was placed in the catheterization room and operated by one laboratory analyst, allowing for immediate reporting of results to the interventional radiologist. During AVS, the radiologist collected blood samples from the right adrenal vein and peripheral vein, immediately handed the samples for RC measurement, and simultaneously proceeded to sample the left adrenal vein. Then, the left-side samples were also measured by the RC assay.

Blood samples of at least 1 mL were collected in VACUETTE^®^ (Kremsmünster, Austria) K3E K3EDTA tubes and centrifuged for 3 min to separate plasma. Due to the assay’s limited measurement range of 1.81–29 µg/dL, samples were diluted before measurement using Cobas Diluent Universal 2 (Roche Diagnostics^®^, Indianapolis, IN, USA). Adrenal vein samples were diluted at a 1:10 ratio, and peripheral samples were diluted at a 1:3 ratio. Subsequently, 50 microliters of diluted plasma samples were added to a tube containing manufacturer-provided detection buffer and mixed thoroughly. Next, 75 microliters of the sample mixture were loaded into the sample well of the designated cartridge and inserted into the i-Chamber incubator (Boditech©, Seoul, Republic of Korea) at 25 °C for 3 min. Then, the cartridge was immediately inserted into the analyzer scanner, and cortisol levels were determined. The time from venous sampling to the result was approximately 10 min. The patient was kept in the catheterization room until the RC results were received from the right and then left vein.

The calculated RC-SI values were used to assess catheterization success during the procedure. We assumed adequate sampling if the RC-SI ratio was above five. However, if the RC-SI ratio was below four, the sampling was deemed unsuccessful, and additional attempts were made until achieving successful sampling or the procedure was deemed unattainable by the operating radiologist. In order to avoid unnecessary resamplings, an SI ratio between 4 and 5 was also considered indicative for adequate sampling if the operating radiologist had confidence in a successful procedure. The threshold of 4 was determined arbitrarily; as of yet, there are no published data on the exact reliability of the RC assay during AVS. It should be noted that when the adrenal cortisol level exceeded the upper measurement range of the assay, even after 1:10 dilution, the calculated RC-SI was reported as “higher than” (>) the ratio between the upper measurement limit and the peripheral cortisol measurement.

Blood samples from the same collection were used for post-procedure laboratory measurements of serum cortisol on the Cobas system and subsequently for plasma aldosterone measurements, if adequate sampling was confirmed by the Cobas assay.

### 2.3. Assays

Measurement of plasma RC concentration was conducted using the iChroma II (Boditech©, Seoul, Republic of Korea) test assay. This immunoassay analyzer measures cortisol levels using europium fluorescent nanoparticle immunoassay technology. The analyzer measures the fluorescence intensity and converts it to a quantitative value, which is displayed as the cortisol level test result. Blood samples of at least 1 mL were collected in VACUETTE^®^ K3E K3EDTA tubes. The method’s performance characteristics, as reported by the manufacturer, were sensitivity of 1.81 μg/dL and intra-assay and inter-assay coefficients of variation of 5.9% and 5.7%, respectively. The measurement range was 1.81–29 μg/dL.

Laboratory serum cortisol was collected in VACUETTE^®^CAT serum separator clot activator tubes. Each tube contained at least 1 mL of blood, and the Cobas e411 analyzer (Roche Diagnostics^®^, Indianapolis, IN, USA) electrochemiluminescence assay was used for cortisol measurement. The method’s performance characteristics, as reported by the manufacturer, were sensitivity of 0.054 μg/dL and intra-assay and inter-assay coefficients of variation of 1.4% and 2.7%, respectively. The measurement range was 0.054–63.4 μg/dL. The laboratory cortisol analysis requires approximately one hour.

Plasma aldosterone was collected during the procedure in VACUETTE^®^ K3E K3EDTA tubes. Each tube cotained at least 1 mL of blood, and measurements were performed by the IDS-iSYS^®^ (Boldon Colliery, UK) aldosterone assay. The method’s sensitivity was 102.5 pmol/L, and intra-assay and inter-assay coefficients of variation were 3.3% and 5.2%, respectively. The measurement range was 102.5–3656 pmol/L. Samples were diluted until an absolute hormone concentration value was observed.

### 2.4. Cost Analysis

Our cost–benefit analysis incorporated the sum of costs associated with implementing the POC-RC method, including the cost of the iChroma analyzer (2685$), the price of each POC-RC test (35$), and the total number of tests used in our cohort. Those were weighted against the expected savings for each repeated AVS session avoided (5000$ per session in our institution) and divided by the number of patients in our cohort.

### 2.5. Statistical Analysis

Sensitivity, specificity, positive, and negative predictive values of RC-SI were calculated using standard methods. Univariate curves of the receiver operating characteristics (ROC) were calculated to define the best cut-off values with relevant sensitivity and specificity for each value. In cases of a ratio result “higher than” a certain value, we used the absolute ratio value for the analysis. Descriptive statistics in terms of mean, standard deviation, median percentiles, and ranges were calculated for all the studied parameters. Normal distribution of the continuous parameters was tested by the Kolmogorov–Smirnov test, and then according to the result, we used the t-test or Mann–Whitney U test for differences between groups. For categorical parameters, we used Pearson chi square. *p* < 0.05 was considered significant. SPSS version 29 was used for all statistical analyses.

## 3. Results

### 3.1. Clinical Characteristics

Between January 2016 and June 2024, a total of 55 AVS procedures were conducted at our institution; of them, 19 procedures (from September 2022) were conducted using RC testing. We compared this RC cohort to a historical control cohort, in which 36 procedures were conducted without RC. Demographic and clinical characteristics of the study groups, as depicted in Table 1, show no statistically significant differences between the groups. Both groups were predominately male (73%), with a mean age of 54 ± 10.9. Both groups of hypertensive patients (baseline systolic blood pressure 153.5 ± 19.5 mmHg) received an average of 2.83 ± 0.98 antihypertensive medications and had a nadir potassium level of 3.15 pmol/L. The patients data is available in Supplementary Materials file (Table S1).

### 3.2. AVS Success Results

The AVS success results are presented in Table 2. In the RC cohort, the success rates were 78.9% and 100% for right and left vein, respectively. While, in the historical cohort, success rates were 66.7% and 97.2% for the right and left vein, respectively. There was a need for more than one sampling attempt in six procedures in the RC cohort and in nine procedures in the historical cohort. There was no significant difference in procedural duration between the two groups. Throughout the entire study period, only one complication was documented—adrenal vein leakage that occurred in a patient in the RC cohort. There was no statistical difference between the number of patients diagnosed with lateralization or referred for surgery between the two groups.

Overall, in six (31.5%) patients in the RC cohort, a low RC-SI value obtained following the use of the RC test enabled real-time clinical decision and led to additional sampling attempts during the same AVS session. In two of those patients (10.5%), resampling resulted in the success of the procedure, while in four patients, the procedure was ultimately unsuccessful despite repeated attempts. Nonetheless, in cases of procedure failure following several sampling efforts, the interventional radiologist concluded that the complex anatomy would hinder success in additional sampling attempts, thereby eliminating the need for repeated AVS sessions.

### 3.3. ROC Curve Analysis

Table 3 presents the RC cohort results. A comparison analysis between SI values obtained from the RC-SI and those obtained from the Cobas standard laboratory SI (LAB-SI) test demonstrated that patients with a LAB-SI > 5 had a significantly higher mean RC-SI value than patients with a lower LAB-SI (*p* < 0.001).

**Figure 1:**
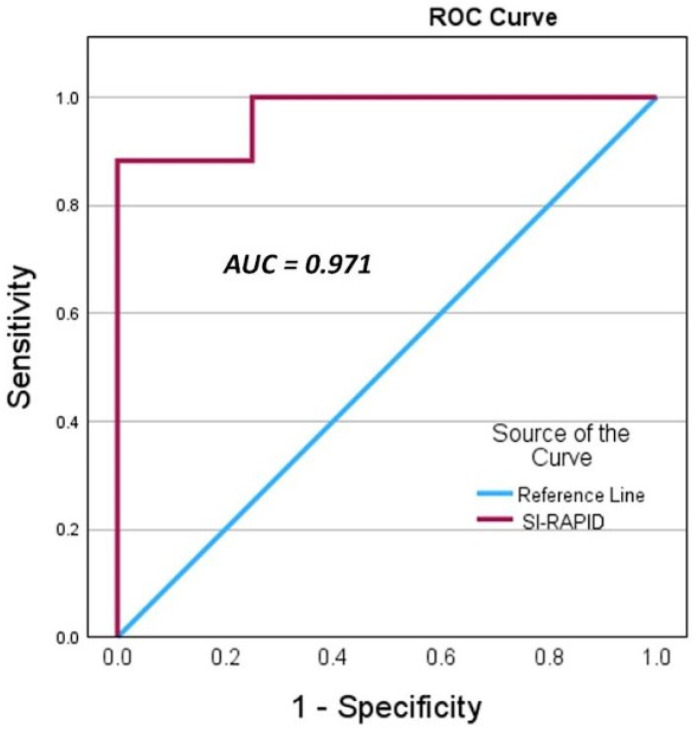
ROC curve analysis of the RC-SI values as predictors of a LAB-SI > 5.

Figure 1 presents the ROC curve analysis of the RC-SI values as predictors of procedural success (LAB-SI > 5). The area under the curve (AUC) is 0.971 (95% CI 0.91–1.03). For an RC-SI threshold value of 2.51, the sensitivity was 100%, and for a threshold value of 4.05, the specificity was 100% (Table 4).

### 3.4. Cost Analysis

We conducted a cost analysis of POC-RC testing implementation in our cohort and demonstrated that the test is economically beneficial, mainly due to savings on repeated AVS sessions. The initial cost of the iChroma device was $2685, and the cost of each measurement was $35. In the RC group, a total of 81 sampling attempts were performed (including repeated attempts)—the average additional cost, including the device cost, was $290 per patient (81 × 35 + 2685)/19. The cost of an AVS procedure is $5000 at our institution. Therefore, in the RC cohort, the use of the rapid test in 19 patients spared 6 patients from potential repeated AVS sessions, resulting in an average saving of $1578 per patient, and considering the additional expenses of the RC test, its use afforded cost savings of an average of $1288 per patient.

## 4. Discussion

In this study, we applied POC-RC measurement during AVS procedures, conducted by a europium fluorescent-based immunoassay analyzer. We found that this tool provided real-time feedback on accurate catheter placement, sparing the need for repeated AVS sessions in 31.5% of cases.

Using POC-RC measurement, AVS success rates increased from 67% to 79% compared with a cohort of AVS procedures performed under similar conditions without the use of rapid cortisol testing, suggesting a role in improving AVS performance. Moreover, the addition of intraprocedural POC-RC did not lengthen average procedure duration significantly, as opposed to reports by others [20], and there was no significant increase in complications.

Several previous studies evaluated the performance of RC-POC measurement with variable results [15,17,18,20,21,22]. In a recent meta-analysis, intraprocedural cortisol measurement was evaluated across 11 studies, including 8 retrospective and 3 prospective studies [19]. Six studies deployed intraprocedural cortisol measurement by rapid transfer of the blood sample to a standard laboratory analyzer, with a result turnaround time between 20 and 120 min. Four other studies employed a POC Quick Cortisol Kit (Trust Medical Corporation, Kobe, Japan), with a 6 min turnaround time, and one study incorporated both methods [19]. Pooled analysis of included studies demonstrated significant improvement in successful bilateral sampling (84% vs. 64%, RR 1.42, 95% CI 1.27–1.59) [19].

To our knowledge, our study is the first to describe the usage of the iChroma^TM^ II test kit in AVS procedures. The test implements a lateral flow competitive immunodetection method utilizing europium fluorescent nanoparticles. The europium nanoparticles offer several advantages over conventional fluorescent labels, such as long fluorescence lifetimes, narrow emission spectra, and remarkable photostability [23]. Former studies on other biomarkers tested by similar iChroma^TM^ II immunoassays found this technique to be fast and easy to use, with adequate analytical performance [24,25].

Implementing the RC method also presents some limitations. The cost of the iChroma device and the measurement kits might be a constraint. However, as demonstrated in our cost analysis, it may prove worthwhile over time by reducing the need for repeated AVS sessions. Additionally, operating the analyzer requires specialized training for laboratory staff, and a laboratory technician must be present throughout the entire procedure.

Compared with other methods, POC-RC assays provide timely results and ease of use. Intraprocedural cortisol measurement by rapid transfer of blood samples to the laboratory can spare the cost of additional analyzers and enable accurate results with well-validated laboratory assays. However, the measurement turnaround time is substantially longer, ranging between 20 and 120 min, compared with only 10 min using the iChroma assay [19]. As patients are typically kept immobile with the catheter sheath in place while awaiting results, the additional time required may increase the risk for procedural complications and also the patient’s discomfort [15,26]. In our study, we found no significant difference in procedural duration using POC-RC compared with the historical cohort.

Another POC-RC assay, described in several previous studies, is the Quick Cortisol Kit (QCA, Trust Medical Corporation, Kobe, Japan). Specifications of size, weight, and measuring range are similar compared with iChroma. Reported QCA measurement turnaround time ranged between 6 and 20 min [18,21,22]. The main difference between the two methods is the immunofluorescence measurement technique; the QCA is based on gold-labeled antibodies, while the iChroma RC is based on europium fluorescent nanoparticles. Studies comparing different immunoassays suggested that europium nanoparticles offered better accuracy over gold-based assays; however, there is no direct head-to-head comparison of the two POC cortisol assays performances [27].

Our cost analyses of POC-RC implementation in AVS procedures revealed that incorporating the RC test resulted in an average cost savings of $1288 per patient, even after accounting for the additional expenses associated with the RC test. This reduction was mainly due to decreased need for repeated AVS sessions. Furthermore, it is expected that the relative savings will increase over time, as the initial investment in the POC device is a one-time expense. Notably, the very high success rates in left-side sampling of close to 100% offer the possibility to use POC-RC only in right-side sampling, thereby decreasing expenses. For example, in the current RC cohort, this would allow a reduced cost of $70 per patient, further increasing the cost savings to $1358 per patient. A recent study from Australia reported an improved diagnostic success rate of AVS from 63% to 94% after implementing intraprocedural RC testing, in addition to affording a net financial saving of AUD ~100 per procedure [20]. The disparity in the amount of savings is probably due to the higher costs of each AVS session in our country, as well as the fact that failed procedures in which repeated sampling was unsuccessful due to challenging anatomy were not accounted for, despite ultimately reducing the need for additional sessions.

Additionally, we created a ROC curve describing RC-SI values as predictors of a LAB-SI above 5 (indicating correct sampling), which yielded an AUC of 0.971. Accordingly, in the RC cohort, for an RC-SI threshold value above 4.05, the specificity and PPV were 100%, sensitivity was 88.2%, and NPV was 50%. Namely, an RC-SI ratio above 4.05 correlated with 100% procedure success as confirmed by laboratory analyzer results, while lower values indicated failed sampling but with less accuracy.

While one of the study’s design strengths is that both historical and RC cohorts underwent AVS procedures by the same interventional radiologist and under similar conditions, it is possible that part of the improvement in AVS success rates can be explained by the interventional radiologist’s increased skills over time. However, this does not account for the decrease in the need for repeated AVS sessions in cases of failed procedures.

The study’s limitations are the retrospective design and usage of a historical control cohort. Additionally, while our results suggested improvement in AVS success rates after the implementation of POC-RC, the differences did not reach statistical significance, likely due to the small sample size. Also, the RC measurement assay has a limited range of values, requiring dilution of the sample at high cortisol levels. As there were no validated instructions for sample dilution in the assay, the dilution applied may affect the test’s accuracy. This issue warrants further investigation in future studies.

With increased detection of PHA, as seen in large screening studies of hypertensive populations [2,3,5], a rise in the demand for AVS procedures is foreseeable. Accurate venous sampling is essential for attaining reliable results, determining if the patient has surgically curable PA, and preventing futile operations. Real-time POC-RC testing is simple and beneficial, and its cost-effectiveness has been demonstrated in the current study and by others [20]. Utilization of POC-RC is now considered a standard practice during AVS at our medical center.

## 5. Conclusions

We demonstrated the effectiveness of utilizing POC-RC measurement during AVS for identifying correct catheter placement in real time, allowing for prompt decisions regarding the need for repeated sampling attempts during the same session. Overall, implementing POC-RC was cost-effective, mainly due to the reduced need for repeated AVS sessions and subsequent costs. Prospective studies with a larger number of cases are needed to validate these conclusions.

## Figures and Tables

**Table 1:** Demographic and clinical characteristics of the study cohorts.

	Total; *n* = 55	Rapid Cortisol; *n* = 19	Historical Control; *n* = 36	*p*-Value
Age (y)	54.0 ± 10.9	54.8 ± 9.8	53.6 ± 11.5	*p* = 0.71
Male (%), female (%)	73%, 27%	79%, 21%	69%, 31%	*p* = 0.54
HTN diagnosis (%)	54 (98%)	19 (100%)	35 (97%)	*p* = 1.00
HTN medications (*n*)	2.83 ± 0.98	2.9 ± 1.05	2.8 ± 0.95	*p* = 0.72
SBP (mm Hg)	153.5 ± 19.5	146.8 ± 19.2	157.4 ± 18.8	*p* = 0.067
DBP (mm Hg)	84.5 ± 13.3	83.9 ± 13.6	84.9 ± 13.4	*p* = 0.79
Serum potassium (mEq/L)–nadir	3.15 ± 0.45	3.05 ± 0.58	3.19 ± 0.39	*p* = 0.34
MRA usage (%)	11 (20.0%)	5 (26.3%)	6 (16.7%)	*p* = 0.48
Aldosterone level (pmol/L)	916.9 ± 565.9	924.8 ± 569.0	912.2 ± 573.2	*p* = 0.94
ARR (pmol/L to microIU/mL) median (IQR)	410 [191–710]	240.6 [153.0–704.6]	432.5 [311.6–835.0]	*p* = 0.093
Adrenal CT findings:				*p* = 0.71
Bilateral	14 (25.9%)	5 (26.3%)	9 (25.7%)	
Right adrenal	10 (18.5%)	5 (26.3%)	5 (14.3%)	
Left adrenal	26 (48.1%)	8 (42.1%)	18 (51.4%)	
Normal adrenals	4 (7.4%)	1 (5.3%)	3 (8.6%)	

HTN, hypertension; SBP, systolic blood pressure; DBP, diastolic blood pressure; MRA, mineralocorticoid receptor antagonists; ARR, aldosterone-to-renin ratio; IQR, interquartile range; mean ± SD.

**Table 2:** Adrenal venous sampling results and outcome: success rates, lateralization, and surgery.

	Total; *n* = 55	Rapid Cortisol; *n* = 19	Historical Control; *n* = 36	*p*-Value
Right Adrenal Vein				
Success rate (%)	39 (70.9%)	15 (78.9%)	24 (66.7%)	*p* = 0.34
Sampling attempts				*p* = 0.87
1	39 (72.2%)	13 (68.4%)	26 (74.3%)	
2	12 (22.2%)	5 (26.3%)	7 (20.0%)	
3	3 (5.6%)	1 (5.3%)	2 (5.7%)	
LAB-SI median (IQR)	18.0 [2.6–28.8]	18.0 [5.2–35.7]	17.6 [0.98–28.0]	*p* = 0.58
Left Adrenal Vein				
Success rate (%)	54 (98.2%)	19 (100%)	35 (97.2%)	*p* = 1.00
1 sampling attempt (%)	55 (100%)	19 (100%)	36 (100%)	NA
LAB-SI median (IQR)	8.5 [6.3–12.4]	10.6 [6.5–15.1]	8.1 [5.85–11.8]	*p* = 0.48
Additional AVS data				
Lateralization (%)	22/37 (59.5%)	9/14 (64.3%)	13/23 (56.5%)	*p* = 0.74
Lateralization side				*p* = 0.86
No lateralization	15 (40.5%)	5 (35.7%)	10 (43.5%)	
Right	3 (8.1%)	1 (7.1%)	2 (8.7%)	
Left	19 (51.4%)	8 (57.1%)	11 (47.8%)	
Lateralization index median (IQR)	7.16 [1.45–30.45]	7.63 [2.05–25.6]	5.8 [1.3–30.9]	*p* = 0.79
Complications (*n*)	1	1	0	
Duration (min)	56.6 ± 25.2	58.06 ± 24.2	54.3 ± 27.6	*p* = 0.69
Surgical treatment (%)	21/53 (39.6%)	7/19 (36.8%)	14/34 (41.2%)	*p* = 0.76

LAB-SI, laboratory selectivity index; IQR, interquartile range.

**Table 3:** Rapid cortisol cohort AVS results.

Case	Gender	Age (y)	CT Scan Findings	Right		Left		Lateralization Side	Lateralization Index	Adrenalectomy (Yes/No)	Adrenal Histology	Notes
				RC-SI *	LAB-SI	RC-SI *	LAB-SI					
1	M	75	Bilateral	>8.25	5.2	>8.25	8	No data	No data	No	NA	
2	F	56	Bilateral	>5.61	24.98	>5.61	15.39	Left	7.16	No	NA	Declined surgical treatment.
3	M	70	Bilateral	>4.2	12.74	>4.32	11.47	No	1.11	No	NA	
4	M	53	Bilateral	>5.37	53.65	>4.83	18.89	No	2.75	No	NA	
5	M	39	Left	>5.04	15.85	>4.88	12.07	Left	3.9	Yes	Adenoma	
6	M	52	Left	>4.98	37.61	>4.75	15.14	Left	40	Yes	Adenoma	
7	M	45	Left	>5.18	37.38	>4.70	12.36	Left	71.6	Yes	Adenoma	
8	F	60	Right	>5.21	18.01	>4.2	5.08	No	1.2	No	NA	
9	M	47	Normal	>4.9	20.71	>4.77	20.15	Left	8.1	Yes	Adenoma	
10	F	58	Left	4.55	7.45	4.26	5.48	Left	20.8	Yes	Adenoma	
11	M	45	Left	>5.46	34.45	5.22	7.72	Left	16	Yes	Hyperplasia	
12	M	49	Right	>6.11	35.74	4.36	6.49	No	1.3	No	NA	
13	M	60	Right	>3.36	19.50	>5.1	6.46	No	2.3	No	NA	
14	M	40	Left	>3.49	62.50	>3.45	18.5	Left	17	Yes	Hyperplasia	
15	F	54	Right	>5.6	16.29	>5.52	6.3	Right	46	No	NA	Planned for surgery
16	M	63	Right	3.91	2.73	5.17	5.38	NA	NA	No	NA	
17	M	61	Left	1.79	0.94	4.81	7.39	NA	NA	No	NA	
18	M	66	Left	2.37	1.15	>4.59	11.01	NA	NA	No	NA	
19	M	48	Bilateral	1.72	0.75	>2.65	10.6	NA	NA	No	NA	

* “RC-SI>” indicates the result was above the assay’s upper limit. Cases in which RC-SI was reported to exceed a value close to our success threshold were regarded as successful, assuming the actual ratio is indeed adequate. RC-SI, rapid cortisol selectivity index; LAB-SI, laboratory selectivity index.

**Table 4:** Metrics of different RC-SI values in predicting successful catheterization (LAB-SI > 5).

RC-SI Value	Sensitivity	Specificity	PPV	NPV
2.51	100% (89.72–100)	75% (19.41–99.37)	97.1% (86.16–99.46)	100% (29.24–100)
4.05	88.2% (72.55–96.70)	100% (39.76–100)	100% (88.43–100)	50% (71.52–28.48)

PPV, positive predictive value; NPV, negative predictive value. Data are expressed as a percent and 95% confidence interval.

## Data Availability

The raw data supporting the conclusions of this article are available from the authors on request.

## Supplementary Materials

The following supporting information can be downloaded at: https://www.mdpi.com/article/10.3390/diagnostics14232692/s1, Table S1: Patients data table.
